# A Manual of Diet in Health and Disease

**Published:** 1875-09

**Authors:** 


### Books Reviewed

---

*A Manual of Diet in Health and Disease.* By Thomas King Chambers, M. D., Oxon., F. R. C. P., London. Philadelphia: Henry C. Lea, 1875. Buffalo: F. Butler & Son.

There seems to be a peculiar charm about the style of Dr. Chambers' writing which adds much to the pleasure of the reader of his works. Even the dryest detail he makes readable and interesting. The volume is divided into three departments: General dietetics, special dietetics in health, and dietetics in sickness. In the preface the author says that his aim has been to make a practical work, and hence all allusion to the chemical and other questions involved is purposely omitted. The portion upon general dietetics treats of the theories of dietetics, the choice of food, its preparation, digestion, and nutrition therefrom derived. These chapters are full of interest, and serve as an introductory to the succeeding section on the special dietetics of health. Commencing with infancy and the regimen of motherhood and infancy, the author proceeds through the stages of childhood and youth, and thence to the various callings and conditions of active life. Under this head also are introduced what he styles hints to the traveler, a chapter on effects of climate, one upon starvation, poverty and fasting, and also upon the decline of life, and one in which the subject of alcohol is considered.

Dr. Chambers differs from the views of Anstie that alcohol, uneliminated, tends to nutrition, and is therefore a food, He, however, thinks that it is of value, and sanctions its use in certain cases when employed with care and judgment. Written by an Englishman for English readers, the work is in some respects not adapted to the habits and characters of the American people, but with slight modifications it will be found to be a most valuable guide to the thoughtful reader, of whatever country.

The closing section of the work, upon the dietetics of disease, embraces nine chapters, a chapter being devoted to each of the large classes of disease, as nervous disorders, inflammations, febrile disorders, consumption, diseases of the circulatory apparatus, etc. Many of the receipes are new, and all are well considered, and seem perfectly adapted to disorders for which they are recommended.

The style of the work makes it valuable to the general as well as to the professional reader, and on the whole it is the best of its kind with which we are acquainted.

